# MuToN Quantifies Binding Affinity Changes upon Protein Mutations by Geometric Deep Learning

**DOI:** 10.1002/advs.202402918

**Published:** 2024-07-12

**Authors:** Pengpai Li, Zhi‐Ping Liu

**Affiliations:** ^1^ Department of Biomedical Engineering School of Control Science and Engineering Shandong University, Jinan Shandong 250061 China

**Keywords:** binding affinity, geometric deep learning, mutation

## Abstract

Assessing changes in protein–protein binding affinity due to mutations helps understanding a wide range of crucial biological processes within cells. Despite significant efforts to create accurate computational models, predicting how mutations affect affinity remains challenging due to the complexity of the biological mechanisms involved. In the present work, a geometric deep learning framework called MuToN is introduced for quantifying protein binding affinity change upon residue mutations. The method, designed with geometric attention networks, is mechanism‐aware. It captures changes in the protein binding interfaces of mutated complexes and assesses the allosteric effects of amino acids. Experimental results highlight MuToN's superiority compared to existing methods. Additionally, MuToN's flexibility and effectiveness are illustrated by its precise predictions of binding affinity changes between SARS‐CoV‐2 variants and the ACE2 complex.

## Graphical abstract

A novel geometric deep learning framework is used for predicting the PPI binding affinity change upon mutations. This approach is biological mechanism‐aware and makes important advances by considering the protein–protein interaction process and allosteric communications. It surpasses SOTA performances and provides a useful tool for understanding the impact of mutations on biological activity.

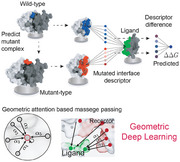

## 1 Introduction

Protein variations affect their functions by altering interactions with proteins or other molecules. Accurate and fast assessing the effects of large number of amino acid replacement due to point mutations helps understanding the cellular activities, which will benefit for revealing the mechanism of complex diseases and targeting drug designs. Quantifying the effects of vast mutation combinations is challenging for resource‐intensive experimental methods.^[^ ^1^ ^]^ Thus, accurate computational methods are urgently needed for effectively annotating mutation effects in high‐throughput scenarios.^[^ ^2^ ^]^

So far, the available computational models for assessing the mutation effects can be generally categorized into two kinds, i.e., unsupervised methods and supervised methods. Benefiting from deep mutational scanning (DMS) database,^[^ ^3^ ^]^ many unsupervised methods leveraged the evolutionary conservation signals for predicting the substitution effects that are experimental measured by the high‐throughput mutational scanning techniques.^[^ ^4^, ^5^, ^6^, ^7^, ^8^, ^9^, ^10^ ^]^ However, these methods typically map arbitrary functions from genotype to fitness, disregarding the diverse range of phenotypic differentiation.^[^ ^11^, ^12^ ^]^

Recently, there have been advancements in supervised methods tailored to directly establish mappings from genetic variants to phenotypic changes, offering precise measurements related to various aspects like thermostability^[^ ^13^, ^14^ ^]^ and ligand‐binding affinity.^[^ ^15^ ^]^ Among them, predicting protein‐protein binding affinity changes affected by variant residues is a crucial aspect of understanding a wide range of biological activities and their relevance to diseases like cancer, such as, identification of driver mutations. The existing methods for this task prediction include mCSM‐PPI2,^[^ ^15^ ^]^ MutaBind2,^[^ ^16^ ^]^ SAAMBE‐SEQ,^[^ ^17^ ^]^ TopNetTree,^[^ ^18^ ^]^ and GeoPPI,^[^ ^19^ ^]^ etc. These methods typically involve utilizing the encoded attributes of wild and mutant residues, along with their neighboring sites, as input, which include various structure‐based, evolutionary‐based and physiochemical‐based features. Subsequently, these attributes are subjected to some regression tasks by traditional machine learning models, such as gradient boosting trees.^[^ ^18^ ^]^ Nonetheless, these approaches fail to account for the genuine physical modifications in protein‐protein interaction (PPI) that result from alterations at the binding site. This limitation is particularly and apparently pronounced when the perturbation affects a region other than the direct binding surface, leading to reduced model prediction performance.^[^ ^18^ ^]^

Geometric deep learning (GDL) focuses on the study of data with an underlying structure that exists within a non‐Euclidean space.^[^ ^20^ ^]^ GDL‐based models have been proved to be effective in some structural biology tasks,^[^ ^21^ ^]^ such as protein structure prediction,^[^ ^22^ ^]^ functional sites identification,^[^ ^23^, ^24^, ^25^ ^]^ PPI interface searching^[^ ^26^, ^27^ ^]^ and rigid protein docking.^[^ ^28^, ^29^, ^30^ ^]^ Regarding specific ways to account for different machine learning tasks, proteins are encoded by: grid,^[^ ^31^ ^]^ surface^[^ ^32^ ^]^ or graph^[^ ^23^ ^]^ representations. With consideration of the intricate and non‐Euclidean characteristics of protein structures, the application of GDL is particularly well‐suited for learning protein structures. When it comes to predicting changes in protein–protein binding affinity induced by mutations, this involves a more complex scenario that entails quantifying alterations in protein binding interfaces and no such GDL methods were performed before.

To address these problems, we introduce here a novel and accurate framework, MuToN, for inferring the protein‐protein binding affinity changes upon Mutations with geometric Transformer Network. MuToN is a mechanisms‐aware framework for modeling protein‐protein binding interface change. The fundamental hypothesis behind MuToN is that alterations in protein binding interface induced by residue mutations lead to changes in its binding affinity. Validated on multiple datasets, MuToN substantially exceeds state‐of‐the‐art (SOTA) methods in quantifying changes in the ΔΔ*G* of protein–protein binding. MuToN operates with high speed and accuracy, enabling the generation of extensive mutational analyzes for protein binding, including application scenarios like virus invasion and mutation. We present a case study using MuToN to predict the binding affinity changes of SARS‐CoV‐2 receptor binding domain (RBD) variants binding with angiotensin converting enzyme 2 (ACE2), resulting a Pearson's correlation coefficient (PCC) of 0.991. In addition, we showcased employing MuToN to accurately characterize the ACE2 binding effect landscape of all amino acid substitutions on the entire RBD sequences.

## 2 Results and Discussion

### 2.1 Overview of MuToN

**Figure 1:**
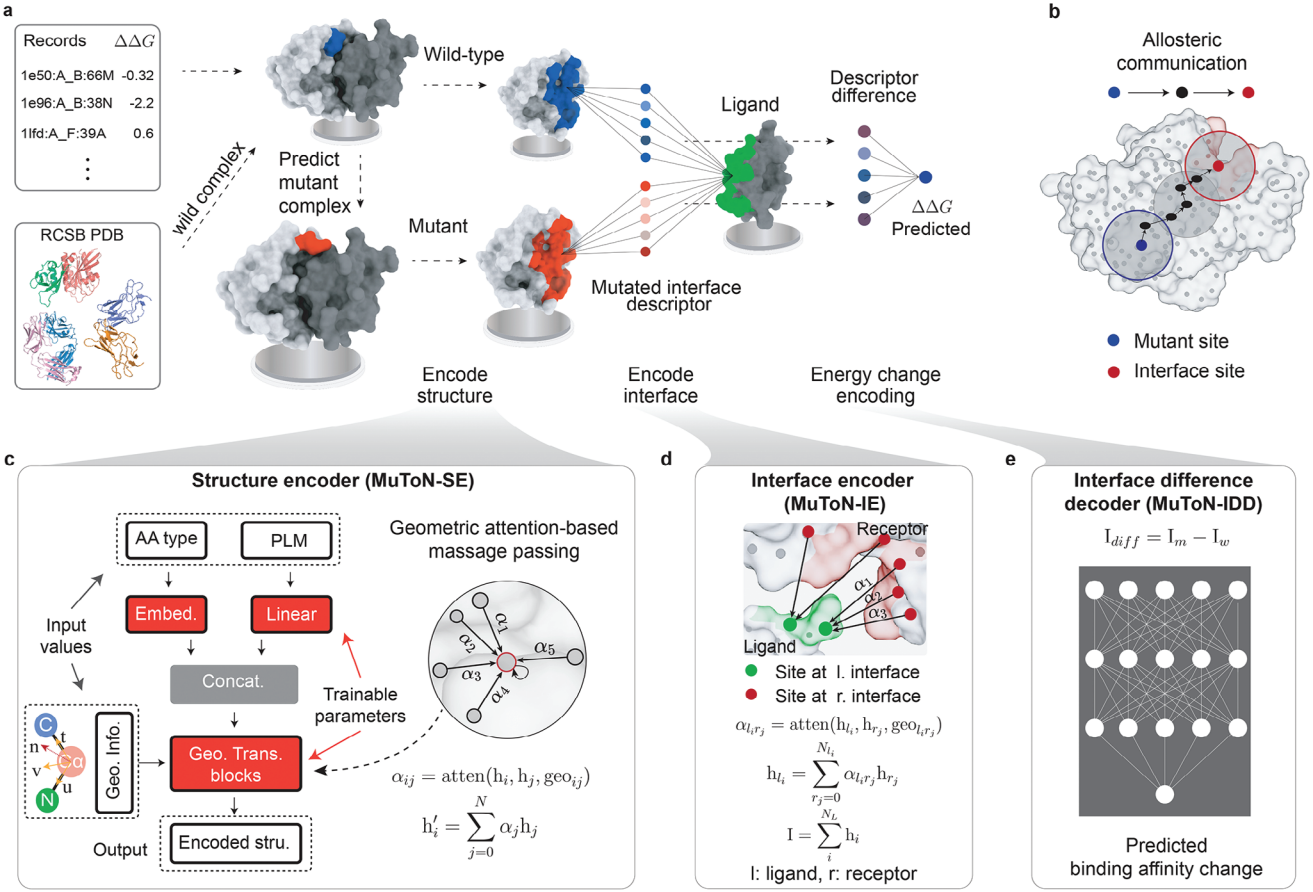
Overflow of MuToN. a) The framework that MuToN predicts protein–protein binding affinity changes induced by amino acid mutation. The wild‐type interaction formations of protein complexes are collected from RCSB PDB database.^[^ ^33^ ^]^ The mutant receptor structures are predicted by computational methods. The structures of wild, mutant receptor as well as the ligand are fed into the structure encoder (MuToN‐SE) for reaching the allosteric site communications. Then the interfaces of wild and mutant complexes are embedded into a feature vector by the interface encoder (MuToN‐IE) module. The feature vector of interface difference between wild‐type and mutant structures are fed into a fully connected neural network to regress the binding affinity changes. b) An schematic diagram for illustrating the allosteric communication of protein sites. We model the mutant effects to ligand binding sites with deep learning by massage passing mechanism. c–e) Relatively details the three core components consisting in MuToN. (c) shows the architecture of protein SE module. The protein nodes are initialized with Amino Acid (AA) types and PLM embeddings, and then updated through Geometric Transformer (Geo. Trans.) blocks. The Geometric Information (Geo. Info.) is fed into each of Geo. Trans. blocks. d) The interface encoder aggregates the features of receptor nodes onto the ligand nodes with a geometric‐attention layer. Then the interface descriptor is represented by averaging the features of ligand residues. e) The interface difference decoder (IDD) module converts the difference in descriptors into a single unit, representing the predicted change in binding affinity. The decoder unit comprises fully connected units without any non‐linear activation functions, a design trick aimed at achieving model antisymmetry.

The global framework of MuToN is shown in **Figure** **1**. MuToN consists of three successive components, i.e., structure encoder (MuToN‐SE), interface encoder (MuToN‐IE), and interface difference decoder (MuToN‐IDD). In the following, we will introduce the three modules in details.

#### 2.1.1 MuToN‐SE

Three protein structures, namely, wild‐type receptor, mutant receptor and ligand are the inputs of the MuToN‐SE module. For simplicity, we refer to the mutated portion of a complex as the “receptor” and the other side as the “ligand”. Each protein structure is represented as a distance‐based, residue‐level graph individually. Nodes in this graph are characterized by one‐hot encoding, which represents elemental residue names and embeddings from a protein language model (PLM). Each node in the graph is associated with a local coordinate reference, determined by the orientations of chemical bonds involving the *C*_α_ atom, nitrogen atom, and carboxyl's carbon atom. This reference system enables message passing throughout the protein structure using geometric transformer blocks, as illustrated in Figure 1c (refer to Experimental Section for more details). The message‐passing module is crucial and should not be overlooked in favor of directly encoding the binding interface. This is particularly important for mutant sites not part of the binding interface but that still affect protein interaction and function. This process, known as allosteric communication in proteins, is depicted in Figure 1b.

#### 2.1.2 MuToN‐IE

MuToN‐SE primarily encodes the states and spatial rearrangements of amino acids for wild‐type receptor, mutant receptor, and ligand. Subsequently, we reassemble the three structures into two protein complexes, wild‐type receptor‐ligand (denoted as *r*_*w*_ − *l*) and mutant receptor‐ligand (*r*_*m*_ − *l*). The MuToN‐IE module processes these two complexes, encoding their binding interfaces into distinct descriptors, respectively denoted as I_*w*_ = Interface(*r*_*w*_, *l*) and I_*m*_ = Interface(*r*_*m*_, *l*). Specifically, given the shared ligand structure between wild‐type and mutant complexes, MuToN‐IE uses a localized geometric attention module to integrate information from the receptor to the ligand side. The resulting fused representations on the ligand are typically sparse, focusing specifically on the ligand's binding interface. Subsequently, a global summation pooling operator is used within MuToN‐IE to derive the interface vectors. The schematic diagram of MuToN‐IE is presented in Figure 1d (refer to Experimental Sectionfor details).

#### 2.1.3 MuToN‐IDD

After using MuToN‐IE, we extract the interface vectors for both wild‐type and mutant complexes. The MuToN‐IDD module is engineered to compress the vector difference into a singular value indicative of the quantitative binding affinity change, represented as ΔΔ*G* = Linear(I_*w*_ − I_*m*_). MuToN‐IDD consists of three fully connected layers, each without bias units and without employing non‐linear activation functions. This configuration instills an antisymmetric property in MuToN, ensuring ΔΔ*G*_*MW*_ = −ΔΔ*G*_*WM*_.

### 2.2 MuToN Quantifies Binding Affinity Changes of PPI Upon Mutations

We utilized the SKEMPI v2.0^[^ ^34^ ^]^ dataset to evaluate MuToN's predictive capabilities. SKEMPI v2.0 encompasses changes in mutation energy at both single and multiple amino acid levels. Existing methods typically train and evaluate single and multiple mutation subsets separately because of limited mutation encoding schemes. Conversely, MuToN analyzes binding interface changes in complexes caused by perturbations, whether they are single or multiple mutations. Benefiting from this approach, we trained and evaluated MuToN on the entire SKEMPI v2.0 dataset. We assessed MuToN's performance using two evaluation schemes based on different dataset splitting strategies: by mutation level and by complex level. In mutation‐level splitting, datasets were divided according to mutation records for training and testing. In complex‐level splitting, each cross‐validated testing dataset contains structures distinct from the training set.

**Figure 2:**
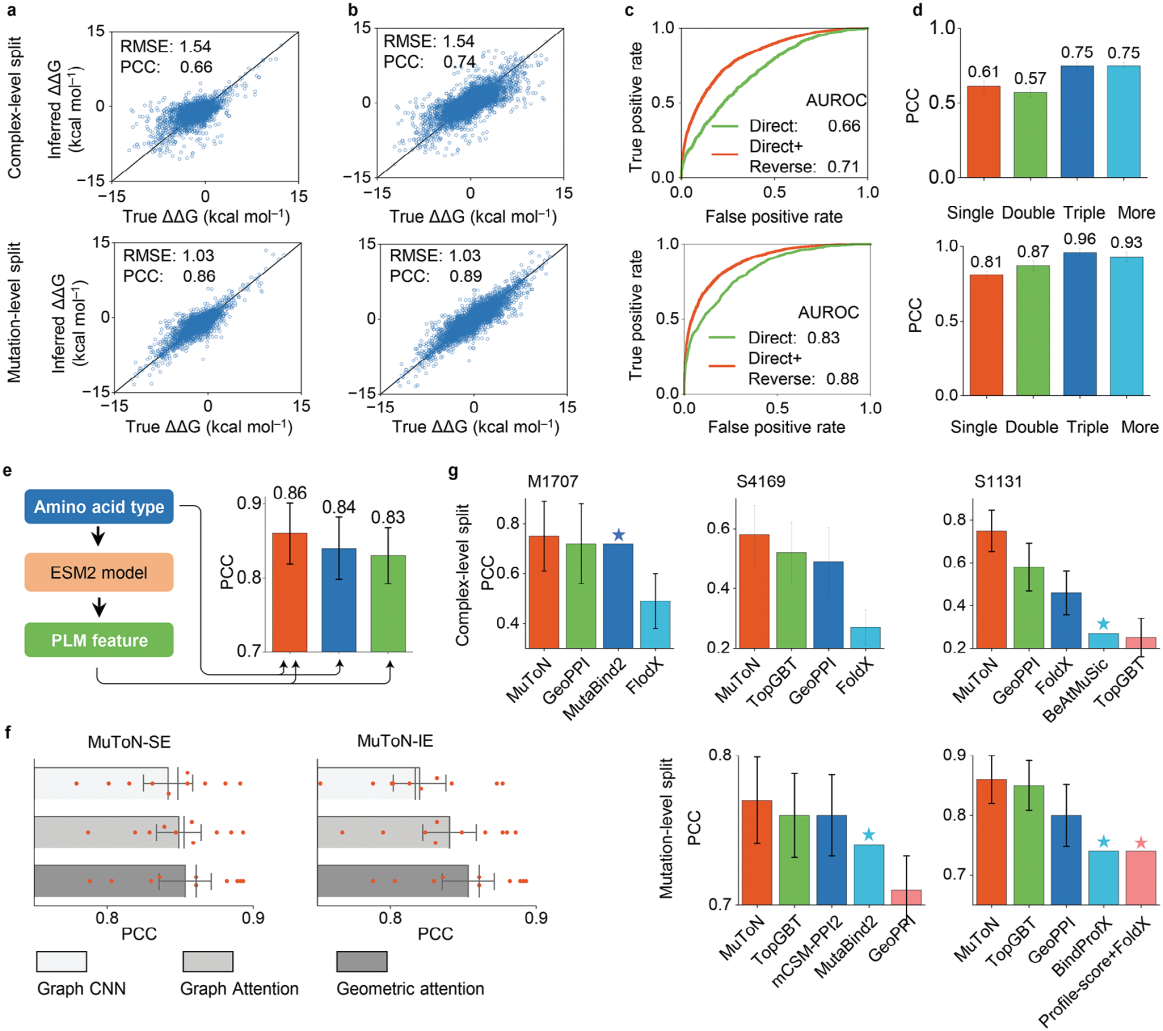
MuToN infers binding affinity change of PPIs upon mutations. In (a–d, g) two distinct training‐testing datasets splitting configurations were utilized to evaluate MuToN: complex‐level splitting and mutation‐level splitting. a) The regression correlation between predicted and experimental values of changes in binding affinity for all mutations in SKEMPI v2.0. b) illustrates the regression correlation between predicted and experimental values of changes in binding affinity for all mutations, including hypothetical reverse mutations in SKEMPI v2.0. c) depicts the Receiver Operating Characteristic (ROC) curves for binary classification of binding affinity changes, distinguishing between increases and decreases in SKEMPI v2.0. d) presents prediction results for varying numbers of mutations. The results are provided with full subset PCCs and standard deviations obtained from tenfold cross‐validation. e) MuToN prediction results by using all features, only amino acid type and only PLM feature. (f) presents the results of an ablation study analyzing various message passing strategies encompassing graph CNN, graph attention, and the geometric attention approach utilized in MuToN. g) Comparison results between MuToN and baseline methods on datasets of M1101, S4169, and S1131. The designation '⋆' means the standard deviation is unavailable.

The evaluations of prediction outcomes are shown in **Figure** **2**a–d. With mutation‐level splitting, MuToN recorded an RMSE of 1.03 and a PCC of 0.86 for predicting changes in binding Gibbs free energy. MuToN exhibits a strong ability to predict anti‐symmetric binding affinities for hypothetical reverse mutations. Notably, without training on reverse mutant samples, MuToN attained a PCC of 0.89 for reverse mutations, as illustrated in Figure 2b. For binary classification of binding affinity changes, MuToN reached a PCC of 0.83 for direct mutations and 0.88 with reverse mutations included in predictions, as shown in Figure 2c.

For scenarios involving multi‐sites mutation, MuToN directly measures the interfacial differences in complex formation before and after mutation occurs, and thus can be applied to multi‐site mutations without requiring model architecture modifications or retraining. MuToN achieved accurate prediction performance for multi‐sites mutations, even surpassing the prediction performance for single‐site mutations, as depicted in Figure 2d. While MuToN demonstrates robust performance in predicting the effects of multi‐site mutations, it is important to acknowledge certain limitations. The number of amino acid mutations that can be effectively analyzed is dependent on the extent of structural and functional perturbations introduced by these mutations. Excessive or highly disruptive mutations may challenge the prediction accuracy, as the underlying model assumes moderate changes to the protein structure caused by residue mutations. Future work will aim to refine these predictive capabilities and address scenarios involving extensive mutations.

We carried out ablation studies to highlight the importance of MuToN's individual modules and components. To assess the input features' significance, we restricted inputs to either amino acid type or PLM features, resulting in PCCs of 0.84 and 0.83, respectively, as shown in Figure 2e. To validate our geometric attention‐based message passing strategy's effectiveness, we replaced it with two alternative aggregation methods: graph convolution and graph attention. Figure 2f illustrates that in experiments replacing MuToN‐SE and MuToN‐IE modules, MuToN, utilizing the geometric attention mechanism, consistently recorded significantly higher PCCs. Additionally, we systematically optimized MuToN's hyperparameters, covering mutant structure computation tools, PLM selection, MuToN‐SE's width and depth, and MuToN‐IE's Gaussian window radius. Details on the hyperparameter optimization process can be found in the Supporting Information MuToN Optimization.

### 2.3 Comparison with SOTA Methods

To benchmark MuToN against SOTA methods, we utilized three widely recognized benchmarks: S4169, S1131, and M1101 for training and evaluation. We employed two dataset splitting strategies alongside tenfold cross‐validation. Comparison results are illustrated in Figure 2g and detailed in Tables S7–S9 (Supporting Information).

#### 2.3.1 Comparison on Dataset S4169

The S4169 dataset comprises all single‐site mutations from SKEMPI v2.0. These mutations occur at arbitrary positions relative to the partner protein. In mutation‐level evaluation, MuToN leads, surpassing the second‐best methods TopGBT and mCSM‐PPI2, with a PCC improvement of approximately 1.3%. In complex‐level evaluation, MuToN significantly outperforms all SOTA methods with an advantage of 13.4% higher in PCC.

#### 2.3.2 Comparison on Dataset S1131

Dataset S1131 curated all single mutations that occur at the partner protein binding interface in SKEMPI v2.0. In mutation‐level assessment, most methods demonstrate accurate predictions. MuToN, TopGBT,^[^ ^18^ ^]^ and GeoPPI^[^ ^19^ ^]^ all achieve a PCC value above 0.8. However, in complex‐level evaluation, where the test set protein structures are entirely unknown to the training set, both TopGBT and GeoPPI show a significant decline. Conversely, MuToN maintains a PCC of 0.7, outperforming the second‐best method, GeoPPI, by about 29% in PCC, establishing it as the leading method.

#### 2.3.3 Comparison on Dataset M1707

The M1707 dataset contains 1,337 records of multiple mutations from SKEMPI v2.0. Including reverse mutations, the dataset expands to 1,707 records across 120 complexes. Comparing MuToN to GeoPPI,^[^ ^19^ ^]^ Discovery Studio,^[^ ^35^ ^]^ and FoldX,^[^ ^36^ ^]^ with a complex‐level dataset splitting strategy for training and testing, MuToN shows roughly 4.2% greater accuracy than the best‐existing method, as measured by PCC.

In summary, MuToN consistently outperforms SOTA methods across various datasets and two data splitting strategies for training and testing. Especially in predictions involving structures completely unknown to the training set, MuToN exhibits remarkable robustness, surpassing other methods.

### 2.4 MuToN Enables Allosteric Communication

**Figure 3:**
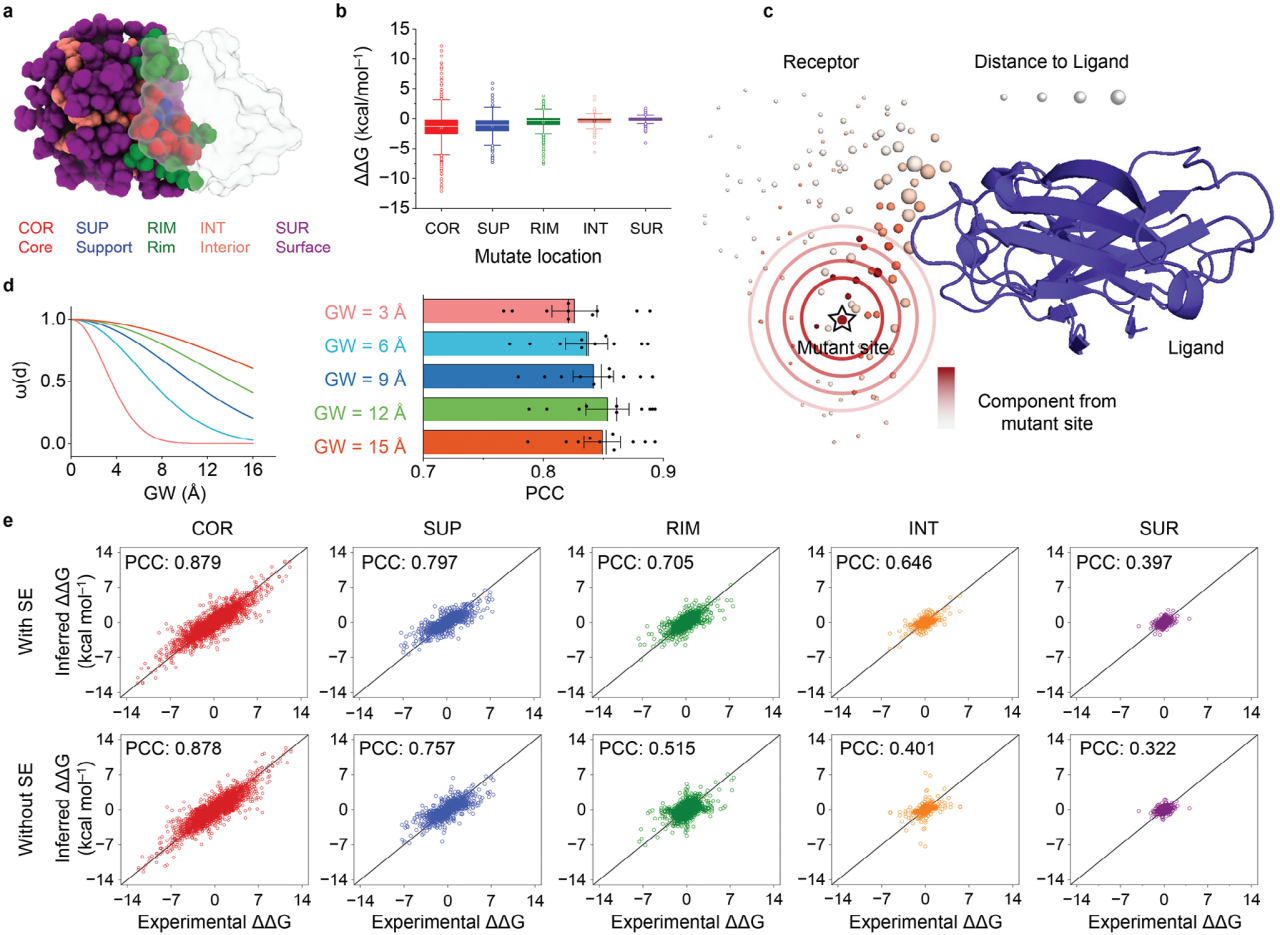
MuToN enables allosteric communication between protein sites. a) Schematic depiction of the structural regions of proteins. b) Distribution of ΔΔ*G* values for different regions in SKEMPI v2.0. c) A case study illustrates the allosteric communication effects recognized by MuToN‐SE module. d) Left, ω(d) is the distance weights which participates the attention of MuToN‐SE.Lines show distance weights v.s. Euclidean distances of sites. Right. A comparison of different GW kernel size on MuToN performance evaluated by mutation‐level splitting. e) Prediction results for different region types with SE module or not show the impacts of this sub‐module on allosteric communication.

Proteins frequently demonstrate an exceptional ability to switch functions, a complex characteristic governed by mechanisms such as molecule binding, covalent changes, or mutations distant from their active sites. This phenomenon, termed as allostery, involves the spatial transmission of information within a protein from one site to another.^[^ ^37^, ^38^, ^39^ ^]^ In our task, sites distant to the binding interface also contribute to the changes in the binding affinity of PPIs. Following Levy's approach,^[^ ^40^ ^]^ we categorized protein regions into five parts: surface, interior, core, rim and support, as illustrated in **Figure** **3**a. The distributions of ΔΔ*G* across different mutation locations in the SKEMPI v2.0 dataset are depicted in Figure 3b. MuToN quantifies the differences in the active interface between wild and mutant complexes. The influence of distant sites on active sites is simulated by a message passing strategy within the MuToN‐SE module.

Figure 3c shows an example of which the INTERLEUKIN‐4 (IL4) binding with its receptor ALPHA chain (PDB ID: 1IAR). The IL4 variant E19A exhibits a threefold increase in binding affinity compared to the wild‐type.^[^ ^41^ ^]^ However, residue E19 is nearly 17 Å away from the receptor in the crystal structure. Using a geometric attention‐based message passing strategy, MuToN‐SE propagates environmental changes near E19 to the IL4‐receptor binding interface. The components received by IL4 sites from E19 are depicted in Figure 3c. We applied a Gaussian window to the weighted distance **d**_*ij*_ to limit the message passing range (as detailed in the Experimental Section). The impact of Guassian window size on MuToN is illustrated in Figure 3d. Choosing a Gaussian window size of 12 Å led to optimal model performance.

Figure 3e depicted the prediction results for different residue types with MuToN. MuToN achieved a PCC value of 0.879 for core region. For other regions, MuToN achieved PCC values ranging from 0.397 to 0.797. The PCC of predictions decreases for regions further from the binding interface. Removing MuToN's SE module significantly reduced its prediction capability, except for mutations in the core region. These results highlight the MuToN‐SE module's effectiveness and efficiency within the MuToN framework, enhancing prediction capabilities through allosteric communication.

### 2.5 MuToN Accurately Predicts ΔΔ*G* of Mutations in the SARS‐CoV‐2 Spike RBD and ACE2

**Figure 4:**
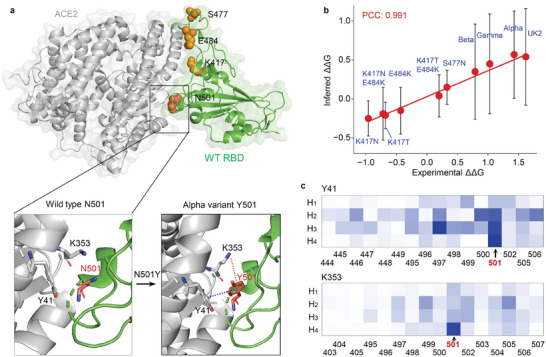
MuToN accurately predicts ΔΔ*G* of mutations in the SARS‐CoV‐two spike RBD and ACE2. a) Overall topology of the SARS‐CoV‐2 spike RBD binding with ACE2. Four common mutations in SARS‐CoV‐2 variants are shown in orange. Structural binding interfaces comparison of wild and N501Y mutation is magnified in the panels. The variant brings two non‐covalent bond to the binding. The cation‐π interaction is colored in orange and the π − π interaction in deep blue. b) plots the MuToN inferred and experimental ΔΔ*G* of ten mutations in the SARS‐CoV‐2 spike RBD and ACE2. Each predicted ΔΔ*G* is given by the mean and standard deviation for the ten models during tenfold‐cross validations. c) The absolute change value in attention weights of the neighboring elements related to ACE2:Y41 and ACE2:K353 during the transition from the wild SARS‐CoV‐2 N501 mutant to the Alpha variant Y501. H1‐H4 relates to the four attention heads in MuToN‐IE module.

To showcase the powerful prediction ability of MuToN, we performed a case study to infer the binding affinity changes between SARS‐CoV‐2 variants and ACE2. A primary mechanism of SARS‐CoV‐2 host cell invasion involves its spike receptor binding domain (RBD) interacting with ACE2. We carried out an analysis to predict the affinity and kinetics effects of five common RBD mutations (namely K417N, K417T, N501Y, E484K, and S477N). The ΔΔ*G* changes in binding affinity for these mutant RBDs with ACE2, including combinations such as the well‐known Alpha, UK2, Beta, and Gamma variants are listed in Table S5 (Supporting Information). As demonstrated in **Figure** **4**b, MuToN accurately forecasts the affinity change trends for the ten variants with ACE2, achieving a PCC of 0.991.

Among the five common RBD mutations, N501Y is particularly notable, as it appears in well‐known variants such as Alpha. N501, located within a loop structure, can be replaced by a tyrosine without affecting protein folding. The N501Y mutation doesn't significantly alter the conformation but enhances affinity with ACE2. This increase in binding affinity results from a new cation‐π interaction with K353 (ACE2) and π − π stacking with Y41 (ACE2),^[^ ^42^ ^]^ as shown in Figure 4a. In this case, the augmented physical interaction significantly influenced the mutated interface vector. Changes in attention weights for ACE2‐Y41 and ACE2‐K353, reflecting aggregated information from neighboring residues, are depicted in Figure 4c. According to MuToN‐IE principles, Y41 and K353 acquire binding features through aggregation from neighboring nodes in the RBD, utilizing attention mechanisms. When the amino acid at position 501 in the RBD changes from N to Y, this substitution has the most significant impact on the attention weights at that position. This example demonstrates MuToN‐IE's efficiency in detecting interface changes and capturing binding interface differences.

### 2.6 MuToN Globally Maps the Effect Landscapes of SARS‐CoV‐2 Mutations

Previously, we utilized MuToN to predict ACE2‐binding affinity changes (ΔΔ*G*) resulting from specific SARS‐CoV‐2 RBD mutations. Understanding how these mutations globally impact the binding landscape could significantly advance our knowledge of evolutionary dynamics and inform vaccine and countermeasure development.^[^ ^43^ ^]^

While the comprehensive kinetics and energetics of mutant SARS‐CoV‐2 RBD binding with ACE2 remain unavailable, the fitness scores of this binding have been measured through DMS techniques.^[^ ^44^ ^]^ We used MuToN which was pre‐trained on SKEMPI for predicting the ΔΔ*G* and assess its correlation with DMS‐measured scores. The Spearman's correlation coefficient between predicted ΔΔ*G*s and DMS scores is 0.18. The moderate correlation may originate from the biophysical ambiguities associated with DMS‐measured binding affinity. For instance, DMS‐measured binding affinity may be affected by various changes in underlying biophysical properties.^[^ ^45^, ^46^ ^]^ Changes in stability, for example, can lead to reduced concentration or altered binding affinity.

**Figure 5:**
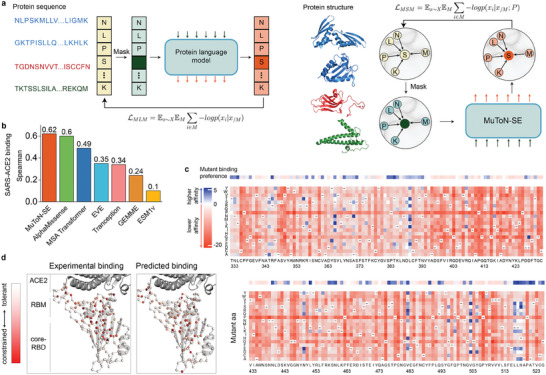
MuToN‐SE maps global binding affinity changes between SARS‐CoV‐2 RBD and ACE2 upon mutations a) Analogous to protein language models, we trained MuToN‐SE on large set of protein structures with position‐conditional masked objects. b) Comparison of MuToN and SOTA methods for predicting the ACE2‐binding fitness effects of single AA substitutions of SARS‐CoV‐2 RBD. The results of comparing methods are obtained from ref. [10]. c) Heatmaps illustrate the predicted ACE2‐binding affinity score of all single mutations in the sequence of Wuhan SARS‐CoV‐2 RBD. Top lines show the corresponding gold standards of mutant binding preference. The mutant binding preference related to each site for individual amino acids with respect to ACE2 binding affinity are obtained from ref. [44] method. d) Experimental and MuToN‐SE predicted mutational constraints of ACE2‐binding mapped onto the SARS‐CoV‐2 RBD structure.

We observed emerging algorithms for predicting mutation effects based on phenotypes, such as AlphaMissense,^[^ ^10^ ^]^ EVE,^[^ ^9^ ^]^ Tranception,^[^ ^47^ ^]^ GEMME,^[^ ^48^ ^]^ MSA Transformer,^[^ ^49^ ^]^ and ESM1v,^[^ ^50^ ^]^ etc. Drawing inspiration from the protein language models among them, we trained MuToN‐SE on large protein structure dataset with position‐conditional amino acid masked objects, as illustrated in **Figure** **5**a. Subsequently, the unsupervised predictor MuToN‐SE was employed to predict the effects of RBD mutations on ACE2 binding affinity. We contextualized the performance of MuToN‐SE in comparison with DMS measured RBD‐ACE2 affinity.^[^ ^44^ ^]^ With approximately 8M trainable parameters, MuToN‐SE achieves a Spearman's correlation coefficient of 0.62, outperforming models based on available sequence information, as shown in Figure 5b. We plot the landscape of ACE2‐binding fitness effects of single amino acid (AA) substitutions of SARS‐CoV‐2 RBD in Figure 5c, d and Figure S1 (Supporting Information), exhibiting strong consistency between MuToN‐SE predictions and experimental measures. The MuToN‐SE parameters and training strategies are detailed in Supporting Information MuToN‐SE optimization.

## 3 Conclusion

In this study, we introduced MuToN for predicting changes in protein‐protein binding affinity caused by amino acid mutations. Unlike existing methods, MuToN accounts for the biological implications, effectively capturing protein‐protein binding mechanisms. This capability enables MuToN to simulate allosteric communication and exhibit inherent antisymmetry in its predictions. MuToN has shown superiority over existing methods across multiple datasets without explicitly detailing physical and chemical features. Moreover, MuToN exhibits strong inductive capabilities in predicting mutations involving unseen tertiary structures, outperforming SOTA methods.

We employed MuToN in a case study to quantify the changes in binding affinity of SARS‐CoV‐2 mutations interacting with ACE2. MuToN accurately predicted the binding affinity trends between ten variants and ACE2, with a PCC of 0.991. Through comparing structural changes between wild‐type and Alpha variant RBDs, we showcased MuToN‐IE's interpretability. Additionally, we trained MuToN‐SE with position‐conditional masked objects, effectively predicting mutation effects identified in DMS studies. Utilizing unsupervised MuToN‐SE, we mapped the mutation effects landscape of SARS‐CoV‐2 RBD binding with ACE2, achieving a Spearman correlation of 0.62.

An important aspect of MuToN is the need for precise variant structures to facilitate subsequent analysis of interface differences. Calculating precise variant structures is a challenging task, especially when multiple sites mutations cause significant structural changes. Additionally, it is difficult to rank and select the most accurate structure from among various variant structures. Although MuToN currently demonstrates relatively robust performance, exploring more accurate variant structure calculation or selection methods will be an important research direction in our future work.

Collectively, we presented an end‐to‐end framework to predict changes in protein binding affinity caused by mutations, leveraging a well‐designed geometric deep leaning network. We have made our data and code open source, facilitating the application of our framework to additional mutation effect quantification challenges.

## 4 Experimental Section

### Datasets Curation

SKEMPI v2.0^[^ ^34^ ^]^ was a database that catalogues changes in binding free energy resulting from amino acid substitutions in well‐structured protein‐protein interactions. This repository included 7,086 records of both single and multiple site mutations. Instances of multiple mutations in different proteins within a complex were excluded. The remaining 5,091 records, which include both single and multiple mutations, were used to train and evaluate the proposed method (available at https://zenodo.org/records/10445253). Additionally, subsets curated by prior studies, including S4169,^[^ ^15^ ^]^ S1131,^[^ ^51^ ^]^ and M1707, were used for comparison with current SOTA methods. Binding affinity was measured using Gibbs free energy (ΔΔ*G*), calculated from the equilibrium dissociation constant (*K*_*D*_) as Δ*G* = *RT*ln *K*_*D*_, with *R* as the ideal gas constant and *T* the temperature in Kelvin. The change in binding affinity due to mutation was calculated as ΔΔ*G*_*w* − *m*_ = Δ*G*_*w*_ − Δ*G*_*m*_.

The ΔΔ*G* values for SARS‐CoV‐2 spike protein receptor binding domain (RBD) variants binding to ACE2 were sourced from Barton et al.^[^ ^52^ ^]^ This dataset covers the effects of common mutations on the binding affinity and kinetics of the RBD/ACE2 interaction. These effects were evaluated using surface plasmon resonance (SPR) measurements. The dataset encompasses a range of variants, including K417N, K417T, S477N, E484K, N501Y (Alpha), K417N+E484K, K417T+E484K, E484K+N501Y (UK2), K417N+E484K+N501Y (Beta), and K417T+E484K+N501Y (Gamma). Details are listed in Table S5 (Supporting Information).

The DMS‐measured binding affinity changes were used for validation of MuToN for mapping the landscapes of SARS‐CoV‐2 RBD variants binding with ACE2. The binding affinities of SARS‐CoV‐2 RBD variants with ACE2 were quantified according to the literature ref. [53].

### Mutant Protein Structure Computing

MuToN necessitates the input of both wild‐type and mutant protein structures. Wild‐type complex structures were sourced from the PDB database.^[^ ^33^ ^]^ However, obtaining mutant protein structures could be challenging due to their limited availability. An attempt was made to apply the three mutant structure tools in the MuToN framework.

1. Modeller.^[^ ^54^ ^]^ Modeller was primarily used for homology or comparative modeling of protein structures. For a site mutation, it outputs a comprehensive view of how a mutation might affect the entire protein structure, including potential changes in *C*_α_ atom positions and overall conformation.
2. FoldX.^[^ ^36^ ^]^ It was a software suite used for protein structure and stability analysis and performs local energy minimization around the mutation site, which means that while side chains and atoms in the immediate vicinity of the mutation may be adjusted, while the overall backbone of the protein generally remains unchanged.
3. ESMFold.^[^ ^55^ ^]^ Additionally, the use of the deep learning‐driven tool ESMFold was explored for predicting mutant structures from mutant sequences.

More details about the choice of the three kinds of mutant structure modeling tools are available in Note S2 (Supporting Information) (Mutant structure modeling tool).

### Protein Structure Encoder (MuToN‐SE)

A geometric transformer architecture was developed to encode protein tertiary structures. MuToN‐SE incorporates four geometric transformer blocks. In each block, node features were updated via geometric‐attention‐based aggregation from neighboring nodes.^[^ ^56^ ^]^ It was determined empirically that the 16 nearest nodes around each node serve effectively as its neighbors, corresponding to an approximate radius of 12 Å around the central node.

The input features correspond to amino acid types (20 possible types) and protein language model (PLM) features. The two kinds of features were relatively encoded into vectors of dimension *N*_*d*_/2, and concatenated into a vector of state size of *N*_*d*_. The reside‐level PLM feature was computed by running ESM2^[^ ^57^ ^]^ against the protein sequence and the dimension was 1280. Considering the spatial information of protein tertiary structure, the protein structure was represented as $P={\left\{x_{i},f_{i}\right\}}_{i=1}^{N}$, where *N* is the number of residues, $x_{i}\in R^{3}$ is the *C*_α_ coordinate of the *i*th residue, *f*_*i*_ is the embedded input feature. The geometric transformer operation was rotation‐invariant for protein structures. To achieve this, a rotation‐equivariant local coordinate frame^[^ ^28^ ^]^ [**n**_*i*_, **u**_*i*_, **v**_*i*_] was established for each residue of the protein. The geometric relation between residue *i* and its neighbors was defined as the concatenation of relative position $R_{ij}\in R^{3}$ and relative orientation $O_{ij}\in R^{9}$,

(1) $$\begin{array}{c} P_{ij}=\left[{\left(x_{j}-x_{i}\right)}^{⊤}\right]\cdot \left[\begin{array}{ccc} n_{i} & u_{i} & v_{i} \end{array}\right] \end{array}$$

(2) $$\begin{array}{c} O_{ij}=\left[{\left(n_{j}-n_{i}\right)}^{⊤};{\left(u_{j}-u_{i}\right)}^{⊤};{\left(v_{j}-v_{i}\right)}^{⊤}\right]\cdot \left[\begin{array}{ccc} n_{i} & u_{i} & v_{i} \end{array}\right] \end{array}$$

resulting in $R_{ij}=\left[P_{ij}∥O_{ij}\right]\in R^{12}$, where ∥ is the concatenation operation and ⊤ represents transposition operation.

A geometric attention‐based aggregation strategy was used for residue feature updating. The hidden representation of residue *i* in the ℓ‐th transformer block is represented as $h_{i}^{\ell }$. The representations of neighbors of residue *i* are first fused with geometric relations:

(3) $$\begin{array}{c} h_{j}^{\ell^{\prime }}=d_{ij}\cdot {\text{MLP}}^{\ell }\left(R_{ij}\right)\cdot h_{j}^{\ell } \end{array}$$

where d_*ij*_ = exp(− ‖**x**_*i*_ − **x**_*j*_‖^2^/2σ^2^) is the Gaussian window smoothed distance between residue *i* and its neighbor residue *j*, MLP is a Multi‐Layer Perceptron containing trainable parameters for geometric relation encoding.

The geometric attention module accepted three types of inputs: the queries (**Q**), the keys (**K**) and the values (**V**). The queries were derived from a non‐linear transformation of the central residue *i*'s representations. Similarly, the keys and values result from non‐linear transformations of the neighboring residues' representations, enriched with geometric relationship information relative to residue *i*:

(4) $$\begin{array}{c} Q_{i}^{\ell }=Q^{\ell }\left(h_{i}^{\ell }\right);\;K_{j}^{\ell }=K^{\ell }\left(h_{j}^{\ell^{\prime }}\right);\;V_{j}^{\ell }=V^{\ell }\left(h_{j}^{\ell^{\prime }}\right) \end{array}$$

where *Q*, *K*, and *V* are feed‐forward neural networks composed of a sequence of linear activation and linear layers. The weights α_*ij*_ for aggregating information from neighboring residues around the central residue *i*:

(5) $$\begin{array}{c} \alpha_{ij}^{\ell }=\text{softmax}\left(Q_{i}^{\ell }{K_{j}^{\ell }}^{T}/\sqrt{d_{\ell }}\right);\;h_{i}^{\ell +1}=\sum_{j\in N_{i}}\alpha_{ij}^{\ell }V_{j}^{\ell } \end{array}$$

where *d*_ℓ_ is the dimension of the ℓth geometric transformer block, representing a scaling factor. The multi‐headed self‐attention mechanism then integrates the outputs from *t* independent attention heads, enabling the capture of diverse geometric interaction patterns:

(6) $$\begin{array}{c} h_{i}^{\ell }\left(\text{MH}\right)=h_{i}^{\ell }\left(1\right)\cdots h_{i}^{\ell }\left(t\right) \end{array}$$

The specifics of the MuToN‐SE module, encompassing quantified hyper‐parameters and the geometric attention algorithm, are detailed in Algorithm S1 (Supporting Information).

### Complex Interface Encoder (MuToN‐IE)

A complex interface encoding network utilizing geometric attention was developed to vectorize the interfaces of both the wild‐type receptor‐ligand, I(*r*_*w*_, *l*), and the mutant receptor‐ligand, I(*r*_*m*_, *l*). Given that the ligand structure remains constant across *r*_*w*_ − *l* and *r*_*m*_ − *l* pairs, the interface encoder specifically maps interactive patterns to ligand sites. For each ligand site, its representation was updated by aggregating information from 16 neighboring receptor sites through geometric attention. This process involved a smooth distance function between ligand and receptor sites, *w*(d) = exp (− d^2^/2σ^2^), where σ denotes the Gaussian window's width, minimizing influence from distant receptor sites. This results in ligand site representations that were sparse and focused on or near the interface. A global summation pooling layer then aggregates these site representations to form the overall interface representation. The geometric attention mechanism in MuToN‐IE mirrors that in MuToN‐SE, with the distinction that central nodes were ligand residues and neighboring nodes were receptor residues. Detailed information about the MuToN‐IE module can be found in Algorithm S2 (Supporting Information).

### Interface Difference Decoder (MuToN‐IDD)

MuToN‐IE produces interface representations for both the wild‐type and mutant protein complexes, represented as I_*w*_ and I_*m*_, respectively. The interface change from the wild‐type to the mutant is quantified by I_*wm*_ = I_*m*_ − I_*w*_. This variation in the interface was then correlated to changes in binding affinity via a linear transformation. The transformation employed three fully connected layers, each devoid of bias units and nonlinear activation functions. This particular configuration preserved the model's antisymmetry characteristic.

### Training and Validation Scheme

To evaluate MuToN's effectiveness, mutation‐level and complex‐level training‐testing dataset splitting strategies were implemented. For mutation‐level splitting, the training and testing datasets were distributed based on distinct mutation instances, which might result in the testing dataset containing samples with underlying structural resemblances to the training dataset. Conversely, in the complex‐level splitting, the structural details of mutations were entirely excluded from the training dataset.

In terms of assessment methodology, a comprehensive training‐validation‐testing cycle embedded was conducted within a tenfold cross‐validation framework. During each cross‐validation fold, 10% of the data was reserved for testing the MuToN model, while the remaining 90% was split in a 9:1 ratio for training and validation.

### Evaluation Metrics

The evaluation metrics used in the main text including Pearson's correlation coefficient (PCC), Spearman's rank correlation coefficient (ρ) and PCC between the direct and the corresponding inverse sequence variations (*r*_*d* − *i*_).

## Conflict of Interest

The authors declare no conflict of interest.

## Supporting information

Supporting Information

## Data Availability

The data that support the findings of this study are openly available in MuToN dataset at 10.5281/zenodo.10445252 , reference number 0.

## References

[1] D. M. Fowler , S. Fields , Nat. Methods 2014, 11, 801. 25075907 10.1038/nmeth.3027 PMC4410700

[2] A. J. Riesselman , J. B. Ingraham , D. S. Marks , Nat. Methods 2018, 15, 816. 30250057 10.1038/s41592-018-0138-4 PMC6693876

[3] D. Esposito , J. Weile , J. Shendure , L. M. Starita , A. T. Papenfuss , F. P. Roth , D. M. Fowler , A. F. Rubin , Genome Biol. 2019, 20, 1. 31679514 10.1186/s13059-019-1845-6 PMC6827219

[4] I. A. Adzhubei , S. Schmidt , L. Peshkin , V. E. Ramensky , A. Gerasimova , P. Bork , A. S. Kondrashov , S. R. Sunyaev , Nat. Methods 2010, 7, 248. 20354512 10.1038/nmeth0410-248 PMC2855889

[5] M. Hecht , Y. Bromberg , B. Rost , BMC Genomics 2015, 16, S1. 10.1186/1471-2164-16-S8-S1 PMC4480835 26110438

[6] Y.‐F. Huang , B. Gulko , A. Siepel , Nat. Genet. 2017, 49, 618. 28288115 10.1038/ng.3810 PMC5395419

[7] M. Kircher , D. M. Witten , P. Jain , B. J. O'roak , G. M. Cooper , J. Shendure , Nat. Genet. 2014, 46, 310. 24487276 10.1038/ng.2892 PMC3992975

[8] P. C. Ng , S. Henikoff , Nucleic Acids Res. 2003, 31, 3812. 12824425 10.1093/nar/gkg509 PMC168916

[9] J. Frazer , P. Notin , M. Dias , A. Gomez , J. K. Min , K. Brock , Y. Gal , D. S. Marks , Nature 2021, 599, 91. 34707284 10.1038/s41586-021-04043-8

[10] J. Cheng , G. Novati , J. Pan , C. Bycroft , A. Žemgulytė, T. Applebaum , A. Pritzel , L. H. Wong , M. Zielinski , T. Sargeant , R. G. Schneider , A. W. Senior , J. Jumper , D. Hassabis , P. Kohli , Ž. Avsec , Science 2023, 381, eadg7492. 37733863 10.1126/science.adg7492

[11] T. A. Hopf , J. B. Ingraham , F. J. Poelwijk , C. P. Schärfe , M. Springer , C. Sander , D. S. Marks , Nat. Biotechnol. 2017, 35, 128. 28092658 10.1038/nbt.3769 PMC5383098

[12] J. Otwinowski , Mol. Biol. Evol. 2018, 35, 2345. 30085303 10.1093/molbev/msy141 PMC6188545

[13] C. Pancotti , S. Benevenuta , G. Birolo , V. Alberini , V. Repetto , T. Sanavia , E. Capriotti , P. Fariselli , Brief. Bioinform. 2022, 23, bbab555. 35021190 10.1093/bib/bbab555 PMC8921618

[14] Y. Chen , H. Lu , N. Zhang , Z. Zhu , S. Wang , M. Li , PLoS Comput. Biol. 2020, 16, e1008543. 33378330 10.1371/journal.pcbi.1008543 PMC7802934

[15] C. H. Rodrigues , Y. Myung , D. E. Pires , D. B. Ascher , Nucleic Acids Res. 2019, 47, W338. 31114883 10.1093/nar/gkz383 PMC6602427

[16] N. Zhang , Y. Chen , H. Lu , F. Zhao , R. V. Alvarez , A. Goncearenco , A. R. Panchenko , M. Li , iScience 2020, 23, 100939. 32169820 10.1016/j.isci.2020.100939 PMC7068639

[17] S. Pahari , G. Li , A. K. Murthy , S. Liang , R. Fragoza , H. Yu , E. Alexov , Int. J. Mol. Sci. 2020, 21, 2563. 32272725 10.3390/ijms21072563 PMC7177817

[18] M. Wang , Z. Cang , G.‐W. Wei , Nat. Mach. Intell. 2020, 2, 116. 34170981 10.1038/s42256-020-0149-6 PMC7223817

[19] X. Liu , Y. Luo , P. Li , S. Song , J. Peng , PLoS Comput. Biol. 2021, 17, e1009284. 34347784 10.1371/journal.pcbi.1009284 PMC8366979

[20] M. M. Bronstein , J. Bruna , Y. LeCun , A. Szlam , P. Vandergheynst , IEEE Signal Process. Mag. 2017, 34, 18.

[21] K. Atz , F. Grisoni , G. Schneider , Nat. Mach. Intell. 2021, 3, 1023.

[22] J. Jumper , R. Evans , A. Pritzel , T. Green , M. Figurnov , O. Ronneberger , K. Tunyasuvunakool , R. Bates , A. Žídek , A. Potapenko , A. Bridgland , C. Meyer , S. A. A. Kohl , A. J. Ballard , A. Cowie , B. Romera‐Paredes , S. Nikolov , R. Jain , J. Adler , T. Back , S. Petersen , D. Reiman , E. Clancy , M. Zielinski , M. Steinegger , M. Pacholska , T. Berghammer , S. Bodenstein , D. Silver , O. Vinyals , et al., Nature 2021, 596, 583. 34265844 10.1038/s41586-021-03819-2 PMC8371605

[23] J. Tubiana , D. Schneidman‐Duhovny , H. J. Wolfson , Nat. Methods 2022, 19, 730. 35637310 10.1038/s41592-022-01490-7

[24] L. F. Krapp , L. A. Abriata , F. Cortés Rodriguez , M. Dal Peraro , Nat. Commun. 2023, 14, 2175. 37072397 10.1038/s41467-023-37701-8 PMC10113261

[25] P. Li , Z.‐P. Liu , Nucleic Acids Res. 2023, 51, e60. 37070217 10.1093/nar/gkad288 PMC10250245

[26] V. Stebliankin , A. Shirali , P. Baral , J. Shi , P. Chapagain , K. Mathee , G. Narasimhan , Nat. Mach. Intell. 2024, 5, 1042.

[27] F. Sverrisson , J. Feydy , B. E. Correia , M. M. Bronstein , in Proceedings of the IEEE/CVF Conference on Computer Vision and Pattern Recognition , IEEE, Piscataway, NJ 2021, pp. 15272–15281.

[28] O.‐E. Ganea , X. Huang , C. Bunne , Y. Bian , R. Barzilay , T. S. Jaakkola , A. Krause , Independent se (3)‐equivariant models for end‐to‐end rigid protein docking[J]. arXiv preprint arXiv:2111.07786, 2021.

[29] H. Stärk , O. Ganea , L. Pattanaik , R. Barzilay , T. Jaakkola , in Proceedings of the 39th International Conference on Machine Learning , PMLR, New York 2022, pp. 20503–20521.

[30] F. Sverrisson , J. Feydy , J. Southern , M. M. Bronstein , B. E. Correia , in International Conference on Learning Representations (ICLR) Machine Learning for Drug Discovery , 2022.

[31] J. Jiménez , S. Doerr , G. Martínez‐Rosell , A. S. Rose , G. De Fabritiis , Bioinformatics 2017, 33, 3036. 28575181 10.1093/bioinformatics/btx350

[32] P. Gainza , F. Sverrisson , F. Monti , E. Rodola , D. Boscaini , M. Bronstein , B. Correia , Nat. Methods 2020, 17, 184. 31819266 10.1038/s41592-019-0666-6

[33] H. M. Berman , J. Westbrook , Z. Feng , G. Gilliland , T. N. Bhat , H. Weissig , I. N. Shindyalov , P. E. Bourne , Nucleic Acids Res. 2000, 28, 235. 10592235 10.1093/nar/28.1.235 PMC102472

[34] J. Jankauskaitė, B. Jiménez‐García , J. Dapkūnas, J. Fernández‐Recio , I. H. Moal , Bioinformatics 2019, 35, 462. 30020414 10.1093/bioinformatics/bty635 PMC6361233

[35] R. Dassault Systèmes BIOVIA , Dassault Systèmes: San Diego 2016.

[36] J. Schymkowitz , J. Borg , F. Stricher , R. Nys , F. Rousseau , L. Serrano , Nucleic Acids Res. 2005, 33, W382. 15980494 10.1093/nar/gki387 PMC1160148

[37] H. N. Motlagh , J. O. Wrabl , J. Li , V. J. Hilser , Nature 2014, 508, 331. 24740064 10.1038/nature13001 PMC4224315

[38] J. Xie , L. Lai , Curr. Opin. Struct. Biol. 2020, 62, 158. 32066080 10.1016/j.sbi.2020.01.011

[39] R. Nussinov , C.‐J. Tsai , Cell 2013, 153, 293. 23582321 10.1016/j.cell.2013.03.034

[40] E. D. Levy , J. Mol. Biol. 2010, 403, 660. 20868694 10.1016/j.jmb.2010.09.028

[41] Y. Wang , B.‐J. Shen , W. Sebald , Proc. Natl. Acad. Sci. 1997, 94, 1657. 9050834 10.1073/pnas.94.5.1657 PMC19972

[42] P. Han , C. Su , Y. Zhang , C. Bai , A. Zheng , C. Qiao , Q. Wang , S. Niu , Q. Chen , Y. Zhang , W. Li , H. Liao , J. Li , Z. Zhang , H. Cho , M. Yang , X. Rong , Y. Hu , N. Huang , J. Yan , Q. Wang , X. Zhao , G. F. Gao , J. Qi , Nat. Commun. 2021, 12, 6103. 34671049 10.1038/s41467-021-26401-w PMC8528823

[43] N. N. Thadani , S. Gurev , P. Notin , N. Youssef , N. J. Rollins , D. Ritter , C. Sander , Y. Gal , D. S. Marks , Nature 2023, 622, 818. 37821700 10.1038/s41586-023-06617-0 PMC10599991

[44] T. N. Starr , A. J. Greaney , S. K. Hilton , D. Ellis , K. H. Crawford , A. S. Dingens , M. J. Navarro , J. E. Bowen , M. A. Tortorici , A. C. Walls , N. P. King , D. Veesler , J. D. Bloom , Cell 2020, 182, 1295. 32841599 10.1016/j.cell.2020.08.012 PMC7418704

[45] X. Li , B. Lehner , Nat. Commun. 2020, 11, 4923. 33004824 10.1038/s41467-020-18694-0 PMC7529754

[46] A. J. Faure , J. Domingo , J. M. Schmiedel , C. Hidalgo‐Carcedo , G. Diss , B. Lehner , Nature 2022, 604, 175. 35388192 10.1038/s41586-022-04586-4

[47] P. Notin , M. Dias , J. Frazer , J. M. Hurtado , A. N. Gomez , D. Marks , Y. Gal , Proc. Mach. Learn. 2022, 162, 16990.

[48] E. Laine , Y. Karami , A. Carbone , Mol.Biol. Evol. 2019, 36, 2604. 31406981 10.1093/molbev/msz179 PMC6805226

[49] R. M. Rao , J. Liu , R. Verkuil , J. Meier , J. Canny , P. Abbeel , T. Sercu , A. Rives , Proc. Mach. Learn. Res. 2021, 139, 8844.

[50] J. Meier , R. Rao , R. Verkuil , J. Liu , T. Sercu , A. Rives , Adv. Neural Inf. Proc. Syst. 2021, 34, 29287.

[51] P. Xiong , C. Zhang , W. Zheng , Y. Zhang , J. Mol. Biol. 2017, 429, 426. 27899282 10.1016/j.jmb.2016.11.022 PMC5963940

[52] M. I. Barton , S. A. MacGowan , M. A. Kutuzov , O. Dushek , G. J. Barton , P. A. Van Der Merwe , eLife 2021, 10, e70658. 34435953 10.7554/eLife.70658 PMC8480977

[53] T. N. Starr , A. J. Greaney , W. W. Hannon , A. N. Loes , K. Hauser , J. R. Dillen , E. Ferri , A. G. Farrell , B. Dadonaite , M. McCallum , K. A. Matreyek , D. Corti , D. Veesler , G. Snell , J. D. Bloom , Science 2022, 377, 420. 35762884 10.1126/science.abo7896 PMC9273037

[54] N. Eswar , B. John , N. Mirkovic , A. Fiser , V. A. Ilyin , U. Pieper , A. C. Stuart , M. A. Marti‐Renom , M. S. Madhusudhan , B. Yerkovich , A. Sali , Nucleic Acids Res. 2003, 31, 3375. 12824331 10.1093/nar/gkg543 PMC168950

[55] Z. Lin , H. Akin , R. Rao , B. Hie , Z. Zhu , W. Lu , N. Smetanin , R. Verkuil , O. Kabeli , Y. Shmueli , A. D. S. Costa , M. Fazel‐Zarandi , T. Sercu , S. Candido , A. Rives , Science 2023, 379, 1123. 36927031 10.1126/science.ade2574

[56] A. Vaswani , N. Shazeer , N. Parmar , J. Uszkoreit , L. Jones , A. N. Gomez , Ł. Kaiser , I. Polosukhin , Adv. neural Inf. Proc. Syst. 2017, 30, 5998.

[57] A. Rives , J. Meier , T. Sercu , S. Goyal , Z. Lin , J. Liu , D. Guo , M. Ott , C. L. Zitnick , J. Ma , R. Fergus , Proc. Natl. Acad. Sci. 2021, 118, e2016239118. 33876751 10.1073/pnas.2016239118 PMC8053943

