# Supplementary material for: MuToN Quantifies Binding Affinity Changes upon Protein Mutations by Geometric Deep Learning
Source: Adv Sci (Weinh). 2024 Jul 12;11(35):2402918. doi: 10.1002/advs.202402918 (PMC11425207; doi:10.1002/advs.202402918)
Supplement: Supplementary file 1 — Supporting Information [file ADVS-11-2402918-s001.pdf]

Advanced Science

Supporting Information for

MuToN quantifies binding affinity changes upon protein mutations by geometric deep learning

Pengpai Li, Zhi-Ping Liu*

***Correspondence: Zhi-Ping Liu.**
E-mail: zpliu@sdu.edu.cn

This PDF file includes:

Supporting text
Figs. S1 to S3
Tables S1 to S9
SI References

Supporting Information Text

1. Supplementary Algorithms

Algorithm 1 Geometric transformer for protein structure encoder

Data: Node feature: $\mathbf{f}_{in} \in \mathbb{R}^{N \times N_d}$,
  Node coordinate: $\mathbf{x} \in \mathbb{R}^{N \times 3}$,
  Node local reference frame: $\mathbf{l} \in \mathbb{R}^{N \times 3 \times 3}$,
  Node neighbors: $\text{nn} \in \mathbb{R}^{N \times M}$
Result: Updated node feature: $\mathbf{f}_{out} \in \mathbb{R}^{N \times N_d}$
$\mathbf{h}_0 = \mathbf{f}_{in}$
for $i \leftarrow 1$ to N_b **do**
  ▷ Sampling node neighbor features and geometric descriptors
  $\mathbf{f}_{nn} \leftarrow \mathbf{h}_i[\text{nn}] \in \mathbb{R}^{N \times M \times N_d}$
  $\mathbf{x}_{nn} \leftarrow \mathbf{x}[\text{nn}] - \mathbf{x}[:, \text{newaxis}, :] \in \mathbb{R}^{N \times M \times 3}$
  $\mathbf{l}_{nn} \leftarrow \mathbf{l}[\text{nn}] \in \mathbb{R}^{N \times M \times 3 \times 3}$
  $\mathbf{dis}_{nn} \leftarrow \exp(-\|\mathbf{x}_{nn}\|^2 / 2\sigma^2) \in \mathbb{R}^{N \times M \times 1}$
  ▷ Assigning neighbor features with geometric descriptors
  $\mathbf{f}_{nn\_pos} \leftarrow \text{matmul}(\mathbf{l}, \mathbf{x}_{nn}) \in \mathbb{R}^{N \times M \times 3}$
  $\mathbf{f}_{nn\_orient} \leftarrow \text{matmul}(\mathbf{l}, \mathbf{l}_{nn}) \in \mathbb{R}^{N \times M \times 9}$
  $\mathbf{f}_{nn\_geo} \leftarrow \text{concat}(\mathbf{f}_{nn\_pos}, \mathbf{f}_{nn\_orient}) \in \mathbb{R}^{N \times M \times 12}$
  $\mathbf{f}_{nn} \leftarrow \mathbf{dis}_{nn} \cdot f_{geo}(\mathbf{f}_{nn\_geo}) \cdot \mathbf{f}_{nn} \in \mathbb{R}^{N \times M \times N_d}$
  ▷ Aggregation by attention
  $\mathbf{Q} \leftarrow f_{query}(\mathbf{h}_i) \in \mathbb{R}^{N \times N_h \times N_d}$
  $\mathbf{K} \leftarrow f_{key}(\mathbf{f}_{nn}) \in \mathbb{R}^{N \times M \times N_d}$
  $\mathbf{V} \leftarrow f_{value}(\mathbf{f}_{nn}) \in \mathbb{R}^{N \times M \times N_d}$
  $\mathbf{h}'_i \leftarrow f_{out}(\text{softmax}(\frac{\mathbf{QK}^T}{\sqrt{Nd}})\mathbf{V}) \in \mathbb{R}^{N \times N_d}$
  ▷ Shortcut and layer normalization
  $\mathbf{h}_{i+1} \leftarrow \text{layernorm}(\mathbf{h}_i + \mathbf{h}'_i)$
$\mathbf{f}_{out} = \mathbf{h}_{N_b+1}$

The characteristic dimensions are the number of residues (N), the dimension of node features ($N_d = 64$), the number of attention heads ($N_h = 4$), the number of geometric transformer blocks ($N_b = 4$) and the number of neighbor residues ($M = 16$). The neighbor hidden states ($\mathbf{f}_{nn}$) of a query residue are embedded with the distance and encoded relative position and orientation to this residue. Thus, the attention area can be localized by using a smooth Gaussian window of radius $\sigma = 12$ Å.

Algorithm 2 Protein complex interface encoder

Data: Node feature: $\mathbf{f}_{r\_in} \in \mathbb{R}^{N_r \times N_d}$, $\mathbf{f}_{l\_in} \in \mathbb{R}^{N_l \times N_d}$
    Node coordinate: $\mathbf{x}_r \in \mathbb{R}^{N_r \times 3}$, $\mathbf{x}_l \in \mathbb{R}^{N_l \times 3}$
    Node local reference frame: $\mathbf{l}_r \in \mathbb{R}^{N_r \times 3 \times 3}$, $\mathbf{l}_l \in \mathbb{R}^{N_l \times 3 \times 3}$
    Nodes of receptor neighboring to ligand: $\mathrm{nn} \in \mathbb{R}^{N_l \times M}$
Result: Updated ligand site features: $\mathbf{f}_{interface} \in \mathbb{R}^{N_l \times N_d}$
▷ Sampling node neighbor features and geometric descriptors
$\mathbf{f}_{nn} \leftarrow \mathbf{f}_{r\_in}[\mathrm{nn}] \in \mathbb{R}^{N_l \times M \times N_d}$
$\mathbf{x}_{nn} \leftarrow \mathbf{x}_r[\mathrm{nn}] - \mathbf{x}_l[:, \mathrm{newaxis}, :] \in \mathbb{R}^{N_l \times M \times 3}$
$\mathbf{l}_{nn} \leftarrow \mathbf{l}_r[\mathrm{nn}] \in \mathbb{R}^{N_l \times M \times 3 \times 3}$
$\mathbf{dis}_{nn} \leftarrow \exp(-\|\mathbf{x}_{nn}\|^2 / 2\sigma^2) \in \mathbb{R}^{N_l \times M \times 1}$
▷ Assigning neighbor features with geometric descriptors
$\mathbf{f}_{nn\_pos} \leftarrow \mathrm{matmul}(\mathbf{l}_l, \mathbf{x}_{nn}) \in \mathbb{R}^{N_l \times M \times 3}$
$\mathbf{f}_{nn\_orient} \leftarrow \mathrm{matmul}(\mathbf{l}_l, \mathbf{l}_{nn}) \in \mathbb{R}^{N_l \times M \times 9}$
$\mathbf{f}_{nn\_geo} \leftarrow \mathrm{concat}(\mathbf{f}_{nn\_pos}, \mathbf{f}_{nn\_orient}) \in \mathbb{R}^{N_l \times M \times 12}$
$\mathbf{f}_{nn} \leftarrow \mathbf{dis}_{nn} \cdot f_{geo}(\mathbf{f}_{nn\_geo}) \cdot \mathbf{f}_{nn} \in \mathbb{R}^{N_l \times M \times N_d}$
▷ Aggregation by quasi-attention
$\mathbf{Q} \leftarrow f_{query}(\mathbf{h}_i) \in \mathbb{R}^{N_l \times N_h \times N_d}$
$\mathbf{K} \leftarrow f_{key}(\mathbf{f}_{nn}) \in \mathbb{R}^{N_l \times M \times N_d}$
$\mathbf{V} \leftarrow f_{value}(\mathbf{f}_{nn}) \in \mathbb{R}^{N_l \times M \times N_d}$
$\mathbf{f}_l \leftarrow f_{out}(\mathbf{Q}\mathbf{K}^T\mathbf{V}) \in \mathbb{R}^{N_l \times N_d}$
$\mathbf{f}_{interface} \leftarrow \mathrm{mean}(\mathbf{f}_l) \in \mathbb{R}^{N_d}$

The characteristic dimensions are the number residues of the receptor and ligand (N_r and N_l), the dimension of node features ($N_d = 64$), the number of attention heads ($N_h = 4$) and the number of neighbor residues ($M = 16$). The neighbor hidden states ($\mathbf{f}_{nn}$) of a query residue on the ligand are embedded with the distance and encoded relative position and orientation to this residue. Thus, the attention area can be localized by using a smooth Gaussian window of radius $\sigma = 6$ Å.

2. MuToN optimization

Experimental setup. We conducted a comprehensive analysis of the proposed MuToN framework, training it on the SKEMPI v2.0 benchmark dataset to identify the optimal settings for performance. For the ablation study, we employed the mutation-level dataset splitting strategy. Each experiment underwent evaluation through a ten-fold cross-validation method. The outcomes were presented using Root Mean Square Error (RMSE) and Pearson's Correlation Coefficient (PCC) metrics on the entire dataset, along with standard deviation values calculated for the ten-fold metrics. In each table, * represents the selected optimal hyperparameter in MuToN.

Mutant structure modeling tool. The MuToN framework relies on the availability of mutant structures, which are currently absent from the PDB database. So far, the computational methods for predicting mutant structures can be categorized into two kinds: homologous modeling and AI-driven structure prediction. For the first kind, we tested Modeller(1) and FoldX(2). For AI-driven structure prediction methods, the most famous AlphaFold(3) is time-consuming and proved to be ineffective for mutational analysis(4). As an alternative, we explored the use of ESMFold(5), which is developed based on a large protein language model.

Table S1. Detailed comparison of mutant protein structure modeling tools.

Mutant tool	RMSE↓	PCC↑
Modeller*	1.032±0.193	0.864±0.041
FoldX	1.046±0.204	0.861±0.042
ESMFold	1.097±0.125	0.844±0.055

For a further discussion about the choice of mutant structure modeling tools, we obtained the distributions of RMSE and GDT-scores between wild-type and computed mutant protein structures, using Modeller, Foldx, and ESMFold, as shown in Fig. S2. It can be seen that the mutant structures calculated by Modeller and FoldX show a very high degree of similarity with the wild-type structures. Especially, the coordinates of the C_α atoms of the mutant amino acids generated by FoldX are totally identical to those of the wild-type. FoldX performs local energy minimization around the mutation site, meaning that while side chains and atoms in the immediate vicinity of the mutation may be adjusted, the overall backbone of the protein, including the C_α atoms, generally remains unchanged. On the other hand, Modeller can generate full atomic models that take into account the entire protein structure. This includes both side chains and the backbone, potentially leading to changes in the positions of C_α atoms if the mutation causes significant structural rearrangements.

Choice of protein language model (PLM). MuToN employs two types of features as inputs: one involves the one-hot encoding of amino acid types, while the other incorporates the embedding of protein language model. Within the framework, a series of Protein Language Model (PLM) models from the ESM project, such as ESM-MSA-1b, ESM-1v, and ESM-2, are utilized. The introduction to these models refers to https://github.com/facebookresearch/esm. Notably, ESM-1v applies an early stopping strategy for parameter selection, aiming to optimize the fitness score of mutational effects across a variety of proteins.

Table S2. Detailed comparisons of PLMs.

ESM models	RMSE↓	PCC↑
ESM-1b	1.056±0.116	0.851±0.031
ESM-1v	1.046±0.122	0.847±0.030
ESM-2*	1.032±0.193	0.864±0.041

Width and depth of MuToN-SE (structure encoder). This comparison involves investigations of the number of geometric transformer blocks and number of units in each block.

Table S3. Detailed comparisons of hyperparameters.

Layer/units	RMSE↓	PCC↑
2/32	1.092±0.186	0.843±0.049
2/64	1.074±0.172	0.846±0.044
4/32	1.078±0.163	0.843±0.042
4/64*	1.032±0.193	0.864±0.041
4/128	1.033±0.207	0.862±0.047
6/64	1.032±0.190	0.863±0.045

Detailed comparisons of smooth Gaussian window of radius.. This comparison involves investigations of the impact of radius of Gaussian window on the performance of MuToN.

Table S4. Detailed comparisons of hyperparameters

Radius of Gaussian window	RMSE↓	PCC↑
7	1.077±0.282	0.847±0.043
6*	1.032±0.193	0.864±0.041
5	1.051±0.176	0.854±0.035
4	1.088±0.118	0.845±0.029
3	1.161±0.196	0.823±0.051
2	1.864±3.331	0.565±0.134

3. MuToN-SE for predicting fitness score

Inspired by protein language models (PLM), for example ESM, we developed a protein structure model based on the framework of MuToN-SE. We trained MuToN-SE through self-supervised learning to capture the intrinsic patterns inherent in the spatial arrangements of amino acids, observed across extensive structure databases. Specifically, in our self-supervised representation learning process, we trained MuToN-SE to identify which amino acids would appropriately fit within specific protein structure environments. Traditionally, the PLMs are trained using masked language modeling (MLM) objective(6):

$$\mathcal{L}_{MLM} = \mathbb{E}_{x\sim X}\mathbb{E}_M \sum_{i\in M} -\log p\left(x_i \mid x_{/M}\right) \quad [1]$$

Similarly, we add the amino acid position condition to MLM objective and obtain the masked structure modeling (MSM) objective:

$$\mathcal{L}_{MSM} = \mathbb{E}_{x\sim X}\mathbb{E}_M \sum_{i\in M} -\log p\left(x_i \mid (x_{/M}, P)\right) \quad [2]$$

where P is the residue spatial information constraint.

We score mutations using the log odds ratio at the mutated position, assuming an additive model when multiple mutations T exist in the same sequence:

$$\sum_{t\in T} \log p(x_t = x_t^{mt} \mid (x_{/T}, P)) - \log p(x_t = x_t^{wt} \mid (x_{/T}, P)) \quad [3]$$

Model hyperparameter. MuToN-SE for masked protein structure model (PSM) consists of 8 geometric transformer blocks and in each the hidden size is 256.

Pretraining dataset. We used the non-redundant PDB90 protein structure dataset to train MuToN-SE. The PDB90 dataset contains about 660k proteins. Limited by the capacity of our computing resource, proteins of length longer than 800 amino acids are eliminated. As a result, 610K proteins participate in the training of MuToN-SE.

Training details. The size of protein is arbitrary to the input of MuToN-SE only if the length of protein is less than 800 amino acids for the limited capacity of GPU memory. The model was optimized using Adam (l_1=0.9, l_2=0.999) with learning rate 10^{-4}. We trained with 128 proteins per batch. Model is evaluated on a subset of deep mutational scans (DMS) collected by Ref.(7). These mutation effect records contain protein sequences and but no available structures. We searched for the available tertiary structures for each sequence. The PDB IDs corresponding to the sequences are listed in Table S6. After each epoch, the model is used to predict the fitness score of the DMS data and output the Spearman's rank correlation coefficient score. If the Spearman's rank correlation coefficient score is not improved for five consecutive epochs, the training process will be terminated.

The prediction capabilities of MuToN-SE and existing methods across 23 different proteins are shown in Supplementary Fig. S3. In these comparisons, AlphaMissense emerged as the overall first-ranked choice (Spearman's correlation of 0.561), while MuToN achieved comparable performance with other methods. Although MuToN-SE did not exhibit superior performance over AlphaMissense, we think that it achieves such good performance with significantly fewer parameters demonstrating the versatility and effectiveness of this sub-module of MuToN.

4. Supplementary Tables

Table S5. $\Delta\Delta G$s for SARS-CoV-2 RBD variants binding to ACE2.

Mutant	$\Delta\Delta G$	Mutant	$\Delta\Delta G$
WT	0	-	-
K417N	-0.96	K417N/E484K	-0.72
K417T	-0.68	K417T/E484K	-0.43
S477N	0.33	E484K/N501Y	1.62
E484K	0.2	K417N/E484K/N501Y	0.79
N501Y	1.43	K417T/E484K/N501Y	1.03

Table S6. PDB IDs corresponding to dataset names of DMS. The dataset names are sourced from Supplementary Table 1 of Ref.(7)

DMS name	PDBs	Reference	PDBs
2020cell	6m0j_E	BLAT_ECOLX_Ranganathan2015	1fqg_A
AMIE_PSEAE_Whitehead	2uxy_A	TIM_SULSO_b0	1igs_A
KKA2_KLEPN_Mikkelsen2014	1nd4_A	DLG4_RAT_Ranganathan2012	1be9_A
UBC9_HUMAN_Roth2017	1u9b_A	BG505_env_Bloom2018	8sw7_A
SUMO1_HUMAN_Roth2017	1wyw_B	GAL4_YEAST_Shendure2015	3coq_A
BLAT_ECOLX_Ostermeier2014	1fgq_A	BLAT_ECOLX_Tenaillon2013	1fgq_A
MTH3_HAEAESTABILIZED_Tawfik2015	1dct_A	PABP_YEAST_Fields2013-singles	6r5k_D
P84126_THETH_b0	1vc4_A	HSP82_YEAST_Bolon2016	2cg9_A
TPMT_HUMAN_Fowler2018	2h11_A	IF1_ECOLI_Kishony	1zo1_W
BRCA1_HUMAN_RING	1jm7_A	BLAT_ECOLX_Palzkill2012	1fgq_A
BG_STRSQ_hmmerbit	1gnx_A	TIM_THEMA_b0	1i4n_A
MK01_HUMAN_Johannessen	4qte_A	PTEN_HUMAN_Fowler2018	1d5r_A
RASH_HUMAN_Kuriyan	5p21_A	RL401_YEAST_Fraser2016	1ubq_A
TPK1_HUMAN_Roth2017	3s4y_A	CALM1_HUMAN_Roth2017	1iwq_A
B3VI55_LIPST_Whitehead2015	4zlu_A		

Table S7. Comparison of SOTA methods for $\Delta\Delta G$ prediction using dataset S4169.

Method	Mutation-level		Complex-level	
	RMSE↓	PCC↑	RMSE↓	PCC↑
MuToN	1.16	0.77	1.52	0.58
GeoPPI[a]	1.25	0.71	1.57	0.49
TopGBT[b]	1.13	0.76	1.47	0.52
mCSM-PPI2[c]	1.19	0.76	-	-
MutaBind2[d]	1.18	0.74	-	-
FoldX[e]	2.73	0.27	2.73	0.27

[a] Prediction metrics are obtained by rerunning the standard alone code at https://github.com/Liuxg16/GeoPPI.
[b] Prediction metrics are obtained by rerunning the standard alone code at https://doi.org/10.24433/CO.0537487.v1.
[c] Prediction metrics are computed from the released data at https://biosig.lab.uq.edu.au/mcsm_ppi2/datasets.
[d] Prediction metrics are sourced from Ref. (8).
[e] Prediction metrics are obtained by rerunning the standard alone code at https://foldxsuite.crg.eu/.

Table S8. Comparison of SOTA methods for $\Delta\Delta G$ prediction using dataset S1131.

Method	Mutation-level		Complex-level	
	RMSE↓	PCC↑	RMSE↓	PCC↑
MuToN	1.26	0.86	1.70	0.75
GeoPPI[a]	1.49	0.80	2.01	0.58
TopGBT[b]	1.30	0.85	2.91	0.25
BindProfX[c]	1.70	0.74	-	-
Profile-score+FoldX[d]	-	0.74	-	-
Profile-score[e]	-	0.67	-	0.27
SAAMBE[f]	-	0.62	-	-
BeAtMuSic[g]	2.46	0.27	2.46	0.27
FoldX[h]	2.18	0.46	2.18	0.46

[a] Prediction metrics are obtained by rerunning the standard alone code at https://github.com/Liuxg16/GeoPPI.
[b] Prediction metrics are obtained by rerunning the standard alone code at https://doi.org/10.24433/CO.0537487.v1.
[c-g] Prediction metrics are obtained by rerunning the standard alone code at https://foldxsuite.crg.eu/.
[h] Prediction metrics are sourced from Ref. (8, 9).

Table S9. Comparison of SOTA methods for $\Delta\Delta G$ prediction using dataset M1707

Method	Mutation-level		Complex-level	
	RMSE↓	PCC↑	RMSE↓	PCC↑
MuToN	1.12	0.88	2.10	0.75
GeoPPI[a]	-	-	2.23	0.72
MutaBind2[b]	-	-	2.25	0.72
FoldX[c]	-	-	3.02	0.49

[a] Prediction metrics are obtained by rerunning the standard alone code at https://github.com/Liuxg16/GeoPPI.
[b] Prediction metrics are sourced from Ref. (9).
[c] Prediction metrics are obtained by rerunning the standard alone code at https://foldxsuite.crg.eu/.

5. Supplementary Figure

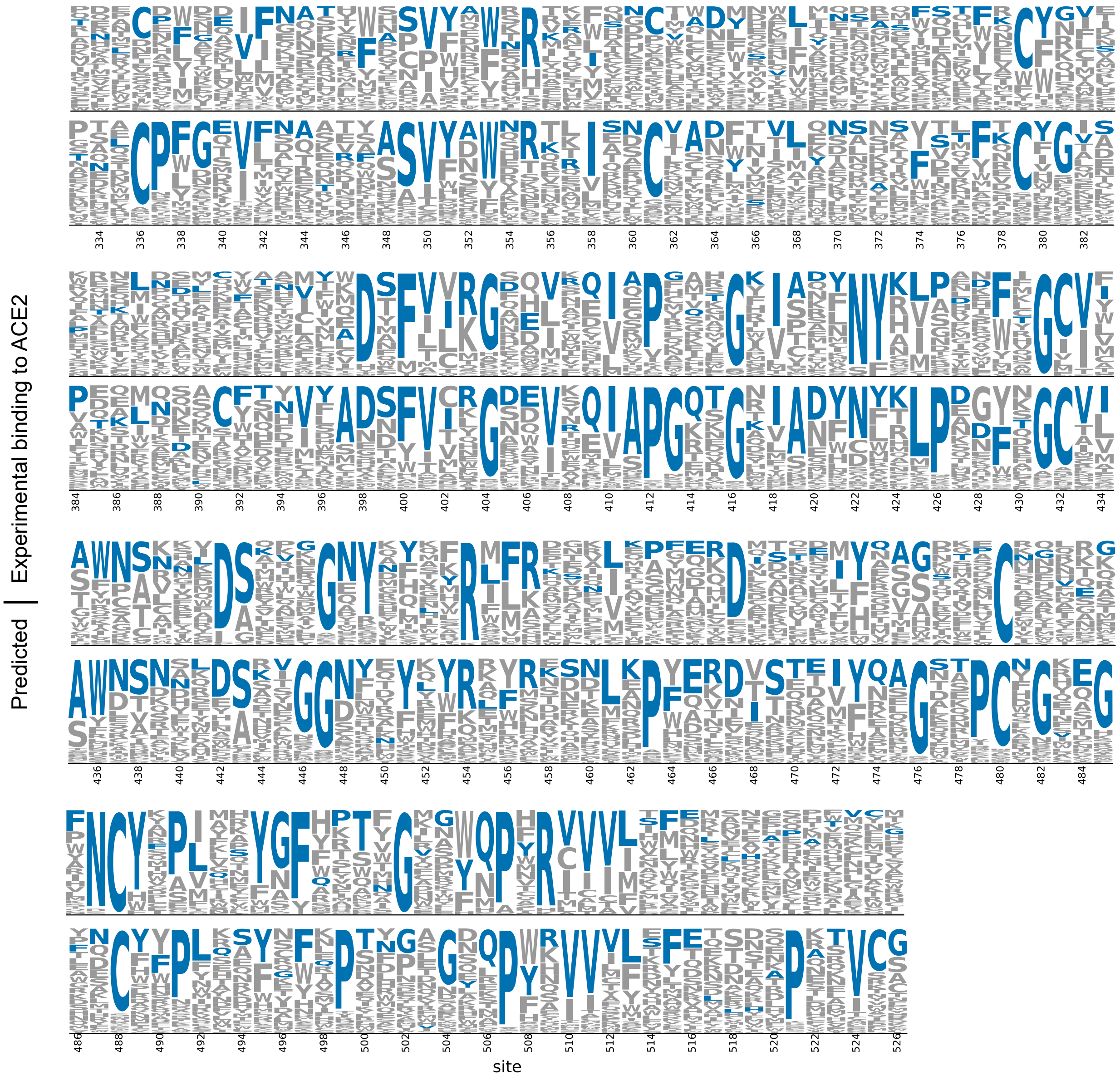

Fig. S1. The sequence logo plot representation showcases the experimental and MuToN-IE predicted mutation effects on binding, corresponding to Figure 5 in the main text. In this figure, the height of each letter signifies the preference of individual amino acids at each site concerning the experimental ACE2 binding (upper row) or the predicted ACE2 binding (lower row). Blue letters denote the wild type of SARS-CoV-2 amino acid residue.

 Pengpai Li, Zhi-Ping Liu*

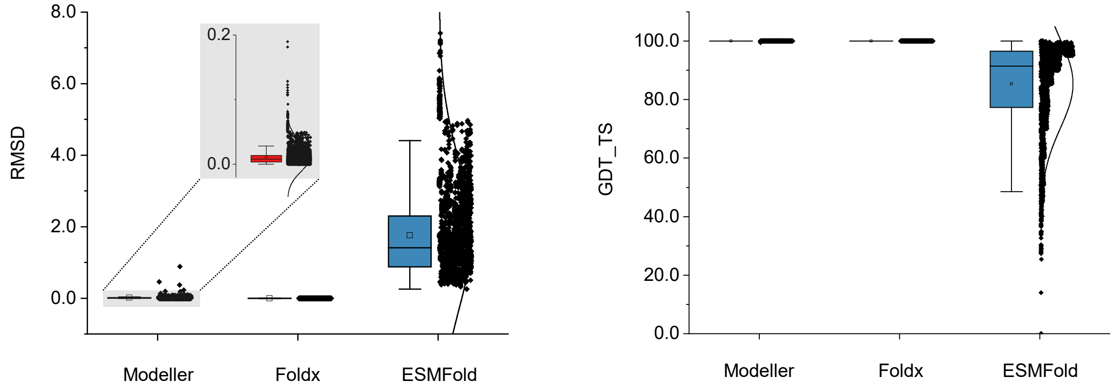

Fig. S2. The distributions of RMSEs and GDT-scores between wild-type and computed mutant protein structures, using Modeller, Foldx, and ESMFold.

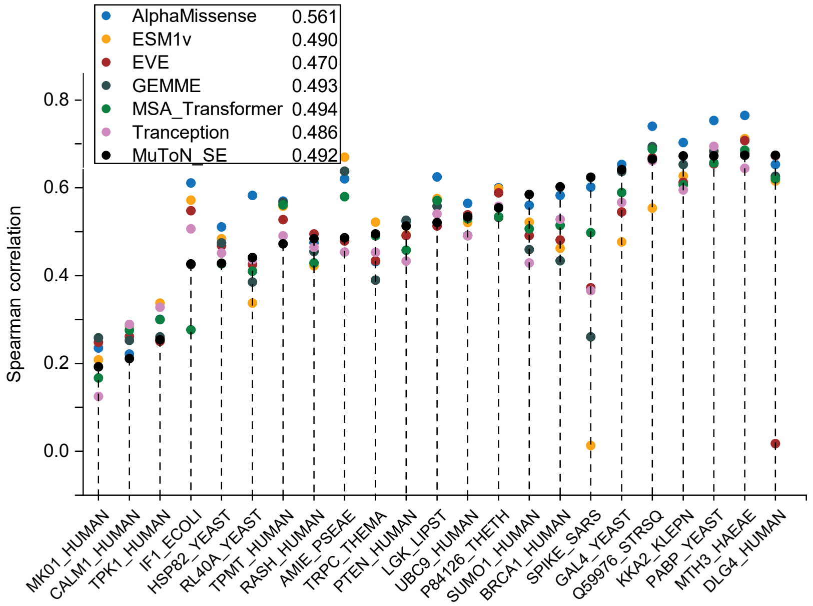

Fig. S3. Per-protein performance of MuToN-SE and SOTA methods. The mean Spearman's correlation of each method is shown in the legend.

References

1. N Eswar, et al., Tools for comparative protein structure modeling and analysis. *Nucleic Acids Res.* **31**, 3375–3380 (2003).
2. J Schymkowitz, et al., The FoldX web server: An online force field. *Nucleic Acids Res.* **33**, W382–W388 (2005).
3. J Jumper, et al., Highly accurate protein structure prediction with AlphaFold. *Nature* **596**, 583–589 (2021).
4. GR Buel, KJ Walters, Can alphafold2 predict the impact of missense mutations on structure? *Nat. Struct. & Mol.* **29**, 1–2 (2022).
5. Z Lin, et al., Evolutionary-scale prediction of atomic-level protein structure with a language model. *Science* **379**, 1123–1130 (2023).
6. A Rives, et al., Biological structure and function emerge from scaling unsupervised learning to 250 million protein sequences. *Proc. Natl. Acad. Sci.* **118**, e2016239118 (2021).
7. AJ Riesselman, JB Ingraham, DS Marks, Deep generative models of genetic variation capture the effects of mutations. *Nat. Methods* **15**, 816–822 (2018).
8. P Xiong, C Zhang, W Zheng, Y Zhang, BindProfX: Assessing mutation-induced binding affinity change by protein interface profiles with pseudo-counts. *J. Mol. Biol.* **429**, 426–434 (2017).
9. X Liu, Y Luo, P Li, S Song, J Peng, Deep geometric representations for modeling effects of mutations on protein-protein binding affinity. *PLoS Comput. Biol.* **17**, e1009284 (2021).
